# Comparative sequence analysis of SARS-CoV-2 suggests its high transmissibility and pathogenicity

**DOI:** 10.2217/fvl-2020-0204

**Published:** 2021-03-08

**Authors:** Kartika Padhan, Mohammad K Parvez, Mohammed S Al-Dosari

**Affiliations:** 1 ^1^Center for Advanced Tissue Imaging, National Institute of Allergy & Infectious Diseases, National Institutes of Health, Bethesda, MD 20892, USA; 2 ^2^Department of Pharmacognosy, College of Pharmacy, King Saud University, Riyadh 11451, Saudi Arabia

**Keywords:** bat-SL-CoV, COVID-19, pathogenesis, SARS-CoV-1, SARS-CoV-2, spike protein

## Abstract

**Aim:** Because the highly pathogenic SARS-CoV-2 is newly introduced to humans, we aimed to understand the unique features of its genome and proteins, crucial for high transmissibility and disease severity. **Materials & methods:** The available genome and protein sequences of SARS-CoV-2 with known human and nonhuman CoV were analyzed using multiple sequence alignment programs. **Results:** Our analysis revealed some unique mutations in SARS-CoV-2 spike, ORF1a/b, ORF3a/3b and ORF8. The most interesting ones were in the spike angiotensin-converting enzyme 2 receptor binding-motif and generation of a furin-like cleavage site as well as deletions of ORF3a ‘diacidic motif’ and the entire ORF3b. **Conclusion:** Our data suggest that SARS-CoV-2 has diverged from SARS-CoV-1 but is most close to bat-SL-CoV. Unique mutations in spike and ORF3a/b proteins strongly endorse its adaptive evolution, enhanced infectivity and severe pathogenesis in humans.

Following the 1918 ‘Spanish Flu’ pandemic, outbreaks of emerging or re-emerging novel viral diseases, such as 1956 ‘Asian Flu’, 1968 ‘Hong Kong Flu’ and 2009 ‘Swine Flu’ have exerted great toll on human health [1]. For centuries, coronaviruses (CoV), the causative agents of common cold have been known to infect humans. Of the known six human CoV (HCoV), the severe acute respiratory syndrome CoV (SARS-CoV-1) originating in China caused the first CoV pandemic during 2002–2003 [2]. Later, a second such pandemic caused by the Middle-East respiratory syndrome CoV (MERS-CoV) originated in Arabian Peninsula during 2012–2013 [2]. Fortunately, most of the countries remained unaffected because both SARS-CoV-1 and MERS-CoV were quickly contained within months.

The ongoing pandemic caused by the novel SARS-CoV-2 disease (COVID-19) that also originated in China is the seventh HCoV infection [3,4]. At the time of writing (February 2021), SARS-CoV-2 has infected over 42 million of the world's population, causing >1 million deaths (https://coronavirus.jhu.edu/map.html). The phylogenetic analysis of SARS-CoV-2 genome has shown its close similarity (96%) with bat-SARS-like CoV (SL-CoV), indicating its zoonosis in horseshoe bats or civet cats [5]. In the absence of specific therapeutics, several repurposed drugs are currently under Phase II/III clinical trials or approved for emergency use [6–8]. Fortunately, of the leading vaccine candidates under final stages of trials, five have been recently granted approval in some countries.

SARS-CoV-2 belongs to the genus Betacoronavirus (Beta-CoV), and has a plus-sense single-strand genomic RNA (∼30 kb) defined into 13 open reading frames (ORF), which code for its structural, nonstructural and accessory proteins [5]. The four structural proteins are crown-like spike (S), envelope (E), membrane (M) and nucleocapsid (N). The S protein has two structural subunits, wherein the ‘S1’ subunit contains the cellular angiotensin-converting enzyme 2 (ACE2) receptor-binding domain (RBD) and the ‘S2’ subunit possesses structural elements required for cell membrane fusion. The M protein is a *trans*-membrane glycoprotein crucial for membrane fusion, whereas the E protein is required for virion assembly and morphogenesis [5]. In case of SARS-CoV-1, the N protein is highly antigenic and is used as a serological marker [9]. The nonstructural replicase proteins (pp1a: nsp1–nsp11 and pp1b: nsp12–nsp16) are involved in mRNA synthesis and replication, and the accessory proteins (3a, 3b, 6, 7a, 7b, 8 and 9b) participate in modulating host innate immunity. Upon infection, SARS-CoV-2 first gets attached to the naso-/oro-pharyngeal inner linings and then moves down to the lungs, which are even richer in ACE2, and triggers cell damage. The bat-SL-CoV S protein is known to bind to civet and horseshoe bat ACE2 receptors [10]. Similarly, SARS-CoV-2 S protein also binds to the ACE2 of airway epithelium, alveolar type-2 pneumocytes that produce pulmonary surfactant [11]. In SARS-CoV-1 patients, the role of apoptosis in lung epithelial cells damage as well as hematological changes including lymphopenia, thrombocytopenia and occasionally leucopenia has been observed, suggesting it role in disease severity [12]. Moreover, the SARS-CoV-1 N, 3a, 3b and 7a proteins are reported to induce apoptosis in cultured cells [13,14].

## Theoretical framework

SARS-CoV-2 has faster ‘human-to-human’ transmission rates and higher pathogenicity than SARS-CoV-1 and MERS-CoV [15]. Notably, the success behind containment of SARS-CoV-1 was due to the fact that the majority of infections happened in hospital setting where spread occurred during late and symptomatic phase [16,17]. Unlike SARS-CoV-1, most of the spread of SARS-CoV-2 is occurring through asymptomatic infection [18], which is a bottleneck for its quick containment. In addition, SARS-CoV-2 has been reported to survive longer than SARS-CoV-1 on certain surfaces including cardboard, plastic and stainless steel [19], which raises the higher chances of its fomite transmission. In general, HCoV do not cause life-threatening disease. However, owing to zoonotic origin of SARS-CoV-1 and SARS-CoV-2, humans lack natural immunity making them aggressively pathogenic [20]. Lacking of this pre-existing immunity, called ‘herd immunity’ is an important reason why naive humans have a much delayed time to develop adaptive immune responses against SARS-CoV-2.

Moreover, SARS-CoV-2 has an incubation period of 2–14 days, which is higher than that of MERS-CoV (2–7 days) and SARS-CoV-1 (2–7 days) [21,22]. The symptoms include fever, cough and breathlessness, which may manifest from mild pneumonia to severe illness [22,23]. Nearly 80% of COVID-19 cases remain asymptomatic or show very mild and self-recovering symptoms and about 15% cases show high fever, pneumonia and breathlessness, whereas up to 5% develop respiratory or multiorgan failure and death [22]. Also, COVID-19 patients with diabetes or hypertension have significantly increased expression of cellular ACE2 receptors, putting them on high risk of mortality [24]. Clinical studies have shown that COVID-19 patients with severe pneumonia may rapidly progress to acute respiratory stress syndrome, septic shock or multiple organ failure and deaths [24]. Nonetheless, unlike SARS-CoV-1 and MERS-CoV, the precise mechanism of modulation of host innate immune responses and severe pathogenesis by SARS-CoV still remains elusive [21]. Moreover, digestive symptom and liver inflammations are also reported in hospitalized COVID-19 patients, which are attributed to cytotoxic T cells and Kupffer cells activities [25–30]. Unlike SARS and MERS cases, cardiac disease, arrhythmia and hypertension have been observed twice as much among COVID-19 critical patients [31,32].

Although, the clinical manifestations of COVID-19 are well understood now, the mechanism(s) underlying its high infection rate and pathogenicity is hitherto not clearly established. Several recent studies have reported comparative sequence analysis revealing some important aspects of specific mutations among SARS-CoV-2 isolates from different geographical regions [33–38]. In this report, we, therefore, analyzed the available human and nonhuman CoV genome and protein sequences to have an insight into its high transmissibility and disease severity.

## Materials & methods

### Data collection

A structured online literature search for peer-reviewed preprint and published articles was conducted on the PubMed, Europe PMC, Medline and Google Scholar portals, using phrases: ‘coronavirus’, ‘SARS-CoV’, ‘MERS-CoV’, ‘SARS-CoV-2’ and ‘bat SARS-like CoV’ as well as ‘COVID-19 infection’, ‘transmission’, ‘pathogenesis’, ‘clinical manifestations’, ‘CoV RNA’ and ‘protein sequences’ etc. The representative genome and protein sequences of various CoV isolates from humans, bats, civets and pangolin were retrieved from NCBI GenBank (www.ncbi.nlm.nih.gov/sars-cov-2) as well as GISAID (www.gisaid.org) database (Table 1).

**Table 1.** List of representative human and nonhuman coronavirus isolates used for sequence alignments.

Species	Coronavirus	Isolates	Year	GenBank accession n
Human	SARS-CoV-2	Wuhan Hu-1	2020	NC_045512.2
	SARS-CoV-2	USA-WA-1	2020	MN985325.1
	SARS-CoV-1	Urbani	2002–2003	AY278741.1
	SARS-CoV-1	Tor2	2002–2003	AY274119.3
	SARS-CoV-1	GD03T10013	2003–2004	AY525636.1
	H-CoV-OC43	UK/London/2011	2011	KU131570.1
	MERS-CoV	EMC-2012	2011	NC_019843.3
Civet	SARS-CoV	SZ3	2003	AY304486.1
	SARS-CoV	Civet007	2004	AY572034.1
Bat	SL-CoV	ZXC21	2015	MG772934.1
	SL-CoV	WIV16	2013	KT444582.1
	SL-CoV	RaTG13	2013	MN996532.1
Pangolin	SL-CoV	MP789	2020	MT084071.1

### Multiple sequence analysis

Translation of protein sequence from cDNA sequence was performed using the sequence analysis application MacVector. Genome and proteins/ORFs sequence alignment was analyzed using multiple sequence alignment program (CLUSTAL W v.10). Mutations were computed with the reference isolate (Wuhan-Hu-1/2019). Percent sequence identity referred to the percentage of matching sequences between isolates.

## Results

### Comparison of genome sequences of SARS-CoV-2 with other CoV

In the present study we compared the genome and protein sequences of SARS-CoV-2, SL-CoV, MERS CoV and other HCoV. Our comparative analysis showed no similarity of SARS-CoV-2 with MERS-CoV and the common cold causing HCoV-OC43. The SARS-CoV sequence from horseshoe bats, civets and humans had strong similarity in RNA sequences (Table 2A). Interestingly, genome analysis of the reference Wuhan Hu-1 and USA WA-1 sequences revealed up to 87 and 96% identity with bat-SL-CoV-ZXC21 and bat-SL-CoV-RaTG13 isolates, respectively. This indicated that SARS-CoV-2 had a separate line of evolution from bat-SL-CoV or bat-like mammals as compared with SARS-CoV-1.

**Table 2.** Comparative sequence analysis of different coronavirus genomes (A) and proteins (B, C and D), showing percent identity between sequences.

A.						
RNA genome	SARS-CoV-2 (Hu-1)	SARS-CoV-1 (Urbani)	SL-CoV (Civet-SZ3)	SL-CoV (Bat-RaTG13)	H-CoV-OC43	MERS-CoV
SARS-CoV-2 Wuhan Hu-1	–	79%	79%	96%	51%	53%
SARS-CoV-1 Urbani	79%	–	99%	94%	50%	53%

B.						
Proteins	Orf1a/1b	Spike (S)	3a	Envelop (E)	Membrane (M)	Nucleocapsid (N)
SARS-CoV-2 *vs* CoV-1	86%	76%	72%	94%	90%	90%

C.						
‘S’ protein	Extracellular domain	Cytoplasmic domain	TM	RBD	RBM	Overall
SARS-CoV-2 *vs* CoV-1	75%	97% (mut: n = 1)	90% (mut: n = 1)	73%	50%	76%

D.						
‘S’ protein	SARS-CoV-1 (2002–2003)	SARS-CoV-1 (2003–2004)	Civet-SL-CoV -SZ3	Bat-SL-CoV- RaTG13	MERS-CoV	H-CoV-OC43
SARS-CoV-2	76%	76%	75%	97%	26%	28%
SARS-CoV-1	–	98%	98%	96%	27%	27%

FP: Fusion peptide; mut: Mutation; RBD: Receptor-binding domain; RBM: Receptor-binding motif; TM: Transmembrane domain.

### Mutational analysis of SARS-CoV-2 S protein

**Figure 1.**
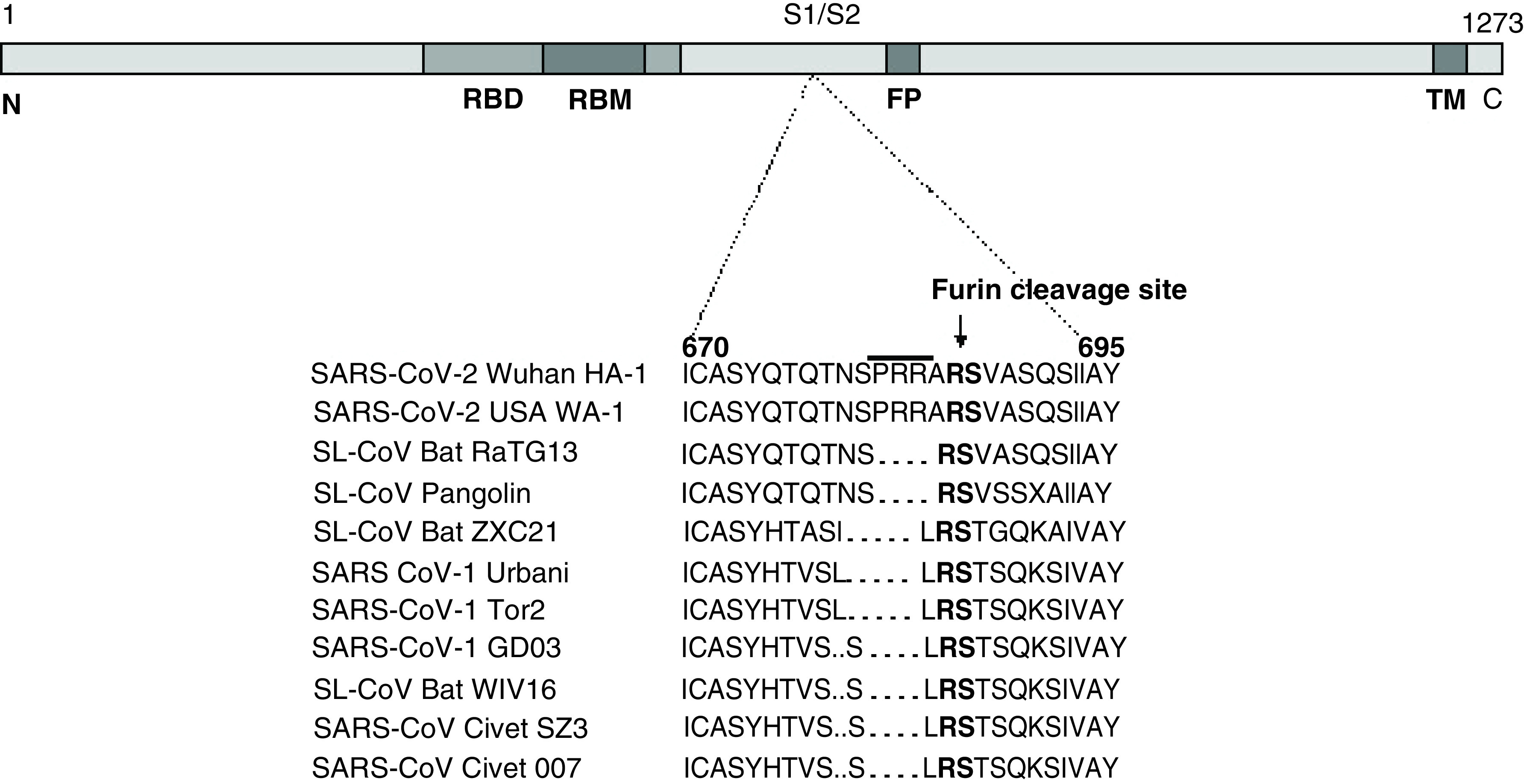
SARS-CoV-2 spike (S) protein. The previously characterized domains include: RBD, RBM, FP and TM. Amino acid sequence (n: 670–695) between various isolates are compared. FP: Fusion peptide; RBD: Receptor-binding domain; RBM: Receptor-binding motif; TM: Transmembrane domain.

Amino acid sequence alignment of SARS-CoV-2 with SARS-CoV-1 showed most mutations acquired in the S protein (Table 2B). Interestingly, the RBM residues located within RBD extracellular domain showed the most variability, suggesting it a ‘hotspot’ of rigorous mutations (Table 2C). Similar to genome sequences, the SARS-CoV-2 S protein had the most identical sequences with those of bat-SL-CoV (Table 2D). One of the unique features of SARS-CoV-2 S protein is the presence of a furin-like cleavage site (Figure 1) with insertions of proline–arginine–arginine–alanine (PRRA) residues, not reported in HCoV or bat-SL-CoV isolates. In line with this, we also did not observe this furin-like cleavage site in any of the known CoV sequence (Figure 1). However, whether the ‘PRRA’ insertion would have evolved in bats or another intermediate mammal, like civet or pangolin remains another area of further investigation.

### Mutational analysis of ORF3a/3b

**Figure 2.**
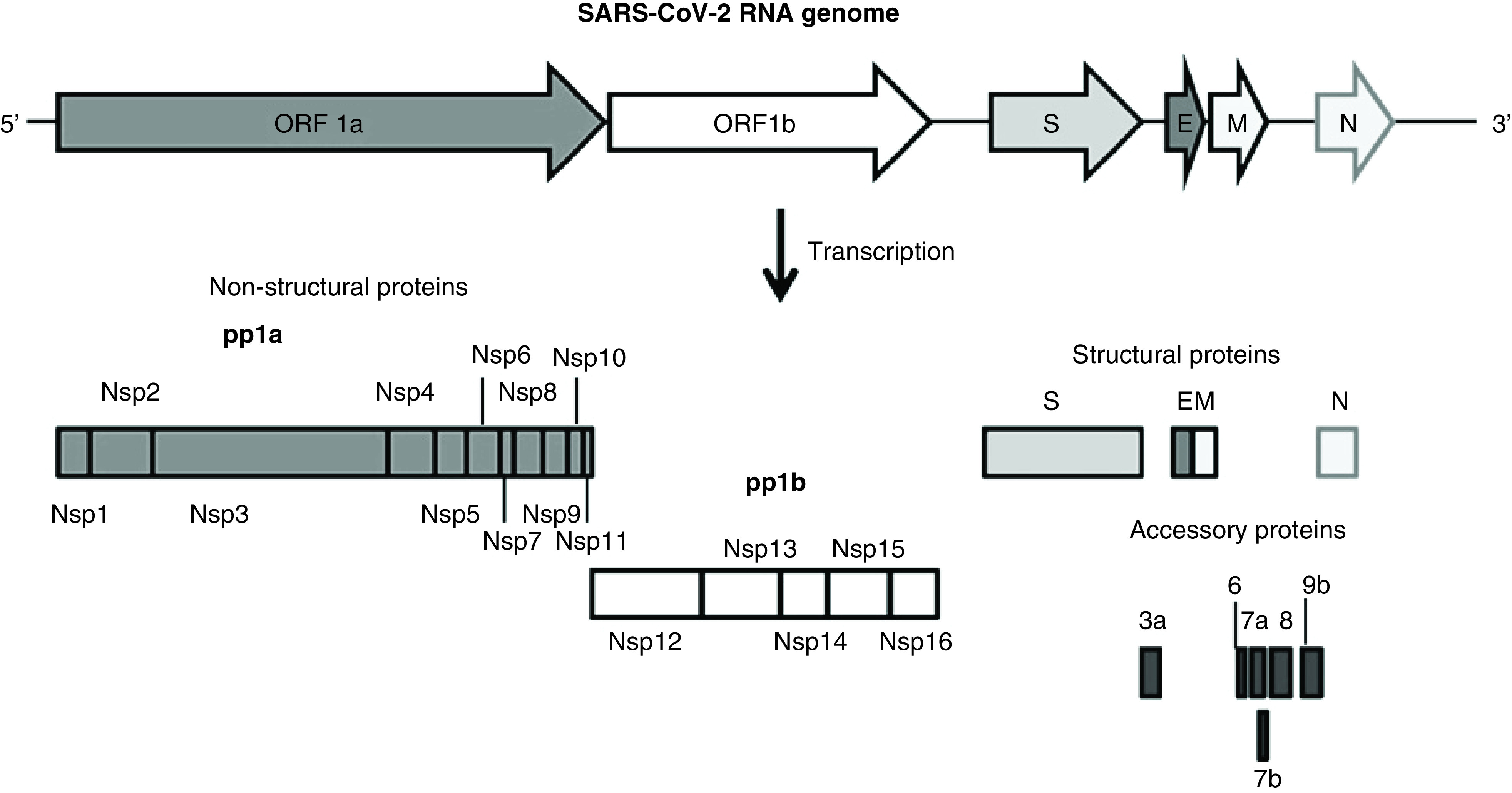
Genome organization of the SARS-CoV-2. The open reading frames (ORF1a and 1b) encoding two large polyproteins, pp1a and pp1b, structural proteins that includes spike **(S)**, envelope **(E)**, membrane **(M)** and nucleocapsid **(N)** and accessory proteins (not drawn to scale). Compare with the accessory protein ‘3b’, which is truncated in SARS-CoV-2.

Unlike other Beta-CoVs, SARS-CoVs express unique ORFs where ORF3a is the largest. Our sequence analysis revealed SARS-CoV-2 encoded 274 residues long 3a as a major mutational ‘hotspot’ with only 72% similarity between SARS CoV-1 (Table 2B). We also observed mutations leading to deletion of the 3a diacidic motif (EXD) in SARS-CoV-2. Notably, a significant truncation in ORF3b (also called orf4 elsewhere) due to introduction of multiple stop codons was also observed. Therefore, the truncated 3b significantly differentiated SARS-CoV-2 from other Beta-CoVs (Figure 2). Taken together, our analysis has highlighted some of the interesting features of SARS-CoV-2 that may be crucial for its pathogenicity and evolution.

## Discussion

Approximately 80% of viruses that infect humans are zoonotic, which are initially ill adapted in a new host, slowly replicated and inefficiently transmitted [39]. Therefore, their ‘animal-to-human’ and ‘human-to-human’ transmission greatly depend on their evolution to virulent strains that can well adapt to human hosts. RNA viruses, due to the high replication-fidelity rate (∼10^-^^4^ error/site/cycle) of their RNA polymerase, are more genetically diversified than DNA viruses [1]. In addition to this, post-transcriptional nucleotide modifications, genetic re-assortment or virus–host recombination may further lead to the establishment of stable strains or lineages in human populations [1]. In a recent analysis, the most prevalent A→G mutation observed in SARS-CoV-2 RNA are suggested to be caused by the host RNA-deamination mechanism [40]. In view of this, both viral RNA replication errors and host RNA-modification system have significant impact on mutation rates toward host adaptation and pathogenicity.

Previously, the phylogenetic analysis has shown very close similarity of SARS-CoV-2 genome (∼96% identity) and S protein (∼80% identity) with bat-SL-CoV (ZXC21 and ZC45) [41–44]. Notably therein, while the ‘S1’ subunit is highly variable (∼70% similarity), ‘S2’ sequences are conserved and shares ∼99% identity with both bat-SL-CoV and SARS-CoV-1 [44,45]. Notably, five critical amino acids in the RBD differ between SARS-CoV-2 and SARS-CoV-1, suggesting strong binding of SARS-CoV-2 S protein with ACE2 receptor and high infectivity [46,47]. Our data showed insignificant similarity of SARS-CoV-2 with MERS-CoV and HCoV-OC43. Interestingly, the two SARS-CoV2 isolates (Wuhan Hu-1 and USA WA-1) revealed up to 87 and 96% sequence identity, respectively with bat-SL-CoV. This indicated that SARS-CoV-2 could have a separate line of evolution from bat-SL-CoV or bat-like mammals (e.g., civet and pangolin) as compared with SARS-CoV-1. Notably, SARS-CoV-1 has been already detected in masked-palm or gem-faced civet cats, which are commonly sold at Chinese wildlife/wet markets [14,48]. In line with this, during the early phase of the SARS-CoV-1 outbreak in China over 40% of the infected individuals were associated with wildlife market or restaurant workers [10]. Likewise, some of the initial cases of SARS-CoV-2 infection were also suggested to be linked to a Wuhan wet market, indicating its possible transmission from bats, civets or pangolins to humans [49]. Our data showed most mutations were acquired in the SARS-CoV-2 S proteins, especially in the RBM, suggesting its enhanced binding to ACE2 as compared with SARS-CoV-1. In addition, we did not observe furin-like cleavage site in any of the known CoV, except in SARS-COV-2, as reported elsewhere [50,51]. Cleavage of SARS-CoV-2 S protein by furin leads to its open conformation that gives advantage of enhanced binding to host ACE2 receptors [51]. Moreover, neuropilin-1 that binds to furin and cleaves substrates has been reported to significantly improve the ACE2-mediated entry of SARS-CoV-2 [52]. Because the role of furin-like proteases in virus entry is a characteristic of pathogenic flu viruses [53], its presence in SARS-CoV-2 strongly supports its high transmission and pathogenicity in humans.

The recent comparative sequence analysis of S, E, M, N and ORF8 proteins of several SARS-CoV-2 isolates of 15 countries with the reference Wuhan Hu-1 has revealed substitutions and/or deletion in S protein at 13 sites, substitutions at three sites in N protein and one substitution in M protein [54]. Another sequence analysis has identified frequent mutations in S, N, ORF1ab and ORF8 proteins in 20 SARS-CoV-2 isolates and evaluated their potential in protein stability and possible functional consequences [34]. Of these, co-occurrences of some mutations across different proteins have suggested their structural and/or functional interactions among other viral proteins, and their involvement in virus adaptability and enhanced transmission. Notably, analysis of structural stability of S protein mutants has further indicated the viability of specific variants that could be more prone to their global distribution, temporally and spatially [34]. Sequence analysis of Russian SARS-CoV-2 isolates, including those from other countries, has revealed a set of seven common mutations in S and N proteins, suggesting their multiple import to Russia, local circulation and varying patterns of spread [36]. Meta-analysis of SARS-CoV-2 isolates within the USA has also identified over 900 unique variants in at least three samples [55]. These included 487 missense and 348 synonymous mutations, four in-frame deletions, five stop codon insertions/deletions and 66 intergenic recombinations. In another study, though diversity of SARS-CoV-2 strains seemed to emerge globally, there was no geographical clustering observed, which suggested their multiple introductions [37]. Interestingly however, their 5’ terminal sequences were more variable as compared with 3’ termini, indicating S, E, ORF1ab and ORF3a as key drivers of diversity, notably RBD as mutational hotspot.

Previous study on SARS-CoV-1 has revealed the presence of a ‘EXD’ motif within the internalization motif of ORF3a that regulates the surface expressions, interaction and internalization of 3a and S proteins [56]. Our sequence analysis has shown mutations leading to deletion of ‘EXD’ in SARS-CoV-2 3a. Notably, the SARS-CoV-2 ORF3b was found to be truncated due to introduction of multiple stop codons, which significantly differentiated it from other Beta-CoVs that code for eight accessory proteins. In line with the reported SARS-CoV-1 truncated 3a activity [57], a recent study has shown the interference of SARS-CoV-2 3b with host interferon system [58]. Because 3a has been reported to modulate IL-2 promoter and interfere with interferon signaling [59], it would be interesting to study the consequence of such observed mutations in clinical settings.

Moreover, SARS-CoV-2 ORF3b and ORF8 has been found to induce a strong antibody response in the early and late phase of infection [60]. In SARS-CoV-2 ORF8, a substitution (Leu→Ser) in 23 isolates has been observed, suggesting their high impact on protein functionality and pathogenesis [37]. Moreover, a proposed phylogenetic tree with representative HCoV and bat-CoV has identified at least two hypervariable hotspots in ORF8 protein, one of which showing a Leu→Ser substitution [33]. Notably, analysis of new SARS-CoV-2 isolates in Italy has reported no evidence for the putative 382-nucleotide deletion in ORF8 as reported in Singapore [38,61]. Taken together, our analysis has endorsed some of the interesting features of SARS-CoV-2 that may be crucial for its pathogenicity and high transmission in humans.

In addition to acquired mutations, differential host factors, such as age, health, physiology, nutritional status, past-exposure, travel history, co-infections, immune-competence, comorbidities and genetics significantly determine the susceptibility to a novel virus [62]. A recent phylogenetic analysis of SARS-CoV-2 sequences from Hong Kong has shown their linkage to European isolates [63]. Interestingly therein, despite insignificant variations between their genomes, they had different clinical presentations, suggesting a more important role of host factors in pathogenicity than mutations. New viruses introduced to humans may further evolve into more aggressive strains as seen for SARS-CoV-2. Herein, the intricate ‘host–pathogen–environment’ interplay is very important in the understanding of the evolution and adaptation of such novel viruses [1]. In view of this, while the emergence of SARS-CoV-2 in naive regions is caused primarily by human movement, local emergence is driven by a combination of environmental and socio-traditional changes [62]. Notably, viral transmission rates are often higher in dense than in sparse populations and social contacts greatly enhance their human-to-human spread. Very interestingly, a recent analysis of SARS-CoV-2 genomic data has revealed its accelerated and high transmission in Italy because of ‘air pollution’ measured with days exceeding the limits set for PM_10_ (particulate matter 10 μm) [64]. In particular, hinterland cities with average set limits along with low wind speed had a very high infection rates compared with coastal cities with high wind speed. This study suggested the accelerated ‘polluted air-to-human’ transmission dynamics of SARS-CoV-2 [64]. Moreover, inhalations of aerosolized or splattered infectious virus particles have been implicated in a community spread. Previously, a study on hundreds of residents of a housing society in Hong Kong showed that the building’s faulty drainage significantly contributed to the aerosolization and respiratory spread of fecal SARS-CoV-1 [65]. In view of this, shedding of infectious SARS-CoV-2 in stool and urine of COVID-19 patients warrants the risk of its ‘waterborne or fecal–oral’ as well as ‘airborne or respiratory’ transmission [66]. Further, the respiratory, flu or pneumonia viruses, including certain HCoV survive in cold seasons and gradually wane with a rise in temperature. Nonetheless, although the emergence of SARS-CoV-2 in colder weather indicated for its plausible seasonality, arrival of summer did not affect the infection rate [62].

The current surveillance of transmission dynamics of infectious pathogens is mainly based on reproduction number (R_0_) and fatality rates, which has been adopted as a real-time monitoring of COVID-19 pandemic [62]. Few mathematical–computational models that combined a framework for host, epidemiological and molecular data for SARS-CoV-2 have demonstrated understanding of patterns of evolution, global spread and country-by-country distribution [67,68]. However, due to the rapid increase in RNA sequencing data, mutation rates differ from viral protein-to-protein and study-to-study. A recently proposed mathematical model has highlighted the emergence phenomena of SARS-CoV-2 and the effects of evolutionary adaptations on spreading processes [69]. Another such model offers quantification (Index c: contagions) of the environmental risk of exposure to future COVID-19 epidemics in a given region [70]. These theoretical models could be helpful in formulating a proactive epidemiological and environmental strategy in the prevention of such pandemics.

## Conclusion

Mutations acquired by SARS-CoV-2 during ‘human-adaptation’ and ‘human-to-human’ spread could provide insights into its transmission dynamics that together with clinical and epidemiological data can predict disease prognosis. In view of this, our genome and protein sequence analysis of SARS-CoV-2 has revealed several novel mutations, the most important ones in the ACE2 receptor binding-motif and generation of a furin-like cleavage site in the spike protein. This suggests high infectivity of SARS-CoV-2 in humans through enhanced cell attachment and facilitated entry. Observed mutations within the replicase protein may be crucial for the enhanced replication of the viral genome. In addition, mutations within the accessory proteins (3a, 3b etc.) could have significant roles in evading or modulating host innate immune system and sustaining virus replication. Nonetheless, the consequence of such mutations on virus infectivity and tissue-tropism remain to be studied in animal models. Since COVID-19 is spread even during the asymptomatic phase of the disease, it will be interesting to study the replication of the virus in early phase and how the innate and adaptive immune system responds to the life cycle of SARS-CoV-2, associated with high transmission and pathogenesis. Nonetheless, a larger sample size, including the recently emerged SARS-CoV-2 mutant strains and a rigorous analysis using more advanced tools would further enhance our knowledge on the subject.

**Summary points**

- The underlying mechanisms of high transmission rate and pathogenicity of SARS-CoV-2 remain poorly understood.
- Our comparative sequence analysis of SARS-CoV-2 and other CoVs identifies unique mutations in spike, ORF1a/b, ORF3a/3b and ORF8.
- Its most conserved E, M, and N protein sequences have, however, undergone fewer mutations.
- The most crucial mutations are in the spike ACE2 binding domain and creation of a furin-like cleavage site as well as deletion of ORF3b.
- Sequence analysis reveals that SARS-CoV-2 has diverged from SARS-CoV-1 but is most close to bat-SL-CoV.
- The roles of unique mutations are, therefore, envisaged in the high transmission and pathogenicity SARS-CoV-2.

## References

[1] Parvez MK, Parveen S. Evolution and emergence of pathogenic viruses: past, present, and future. Intervirology 60(1-2), 1–7 (2017). 28772262 10.1159/000478729 PMC7179518

[2] WHO. Summary of probable SARS cases with onset of illness. 1 November 2002 to 31 July 2003. www.who.int/csr/sars/country/table2004_04_21/en/

[3] Ren LL, Wang YM, Wu ZQ Identification of a novel coronavirus causing severe pneumonia in human: a descriptive study. Chin. Med. J. (Engl). 133(9), 1015–1024 (2020). 32004165 10.1097/CM9.0000000000000722 PMC7147275

[4] Wu F, Zhao S, Yu B A new coronavirus associated with human respiratory disease in China. Nature 579, 265–269 (2020). 32015508 10.1038/s41586-020-2008-3 PMC7094943

[5] Chen Y, Liu Q, Guo D. Emerging coronaviruses: genome structure, replication, and pathogenesis. J. Med. Virol. 92(4), 418–423 (2020). 31967327 10.1002/jmv.25681 PMC7167049

[6] Grein J, Ohmagari N, Shin D Compassionate use of remdesivir for patients with severe Covid-19. N. Engl. J. Med. 382(24), 2327–2336 (2020). 32275812 10.1056/NEJMoa2007016 PMC7169476

[7] Jeyanathan M, Afkhami S, Smaill F, Miller MS, Litchy BD, Xing Z. Immunological considerations for COVID-19 vaccine strategies. Nat. Rev. Immunol. 20(10), 615–632 (2020). 32887954 10.1038/s41577-020-00434-6 PMC7472682

[8] Parvez MK, Padhan K. Current advances in novel SARS-CoV-2 disease (COVID-19) treatment and intervention strategies. Coronaviruses 2, 1–6 (2021).

[9] Leung DT, Tam FC, Ma CH Antibody response of patients with severe acute respiratory syndrome (SARS) targets the viral nucleocapsid. J. Infect. Dis. 190(2), 379–386 (2004). 15216476 10.1086/422040 PMC7110057

[10] Ge XY, Li JL, Yang XL Isolation and characterization of a bat SARS-like coronavirus that uses the ACE2 receptor. Nature 503(7477), 535–538 (2013). 24172901 10.1038/nature12711 PMC5389864

[11] Li W, Moore MJ, Vasilieva N Angiotensin-converting enzyme 2 is a functional receptor for the SARS coronavirus. Nature 426(6965), 450–454 (2003). 14647384 10.1038/nature02145 PMC7095016

[12] Tan L, Wang Q, Zhang D Lymphopenia predicts disease severity of COVID-19: a descriptive and predictive study. Signal Transduct. Target Ther. 5(1), 33 (2020). 32296069 10.1038/s41392-020-0148-4 PMC7100419

[13] Padhan K, Minakshi R, Towheed MAB, Jameel S. Severe acute respiratory syndrome coronavirus 3a protein activates the mitochondrial death pathway through p38 MAP kinase activation. J. Gen. Virol. 89(Pt 8), 1960–1969 (2008). 18632968 10.1099/vir.0.83665-0

[14] Tan YJ, Lim SG, Hong W. Regulation of cell death during infection by the severe acute respiratory syndrome coronavirus and other coronaviruses. Cell Microbiol. 9(11), 2552–2561 (2007). 17714515 10.1111/j.1462-5822.2007.01034.x PMC7162196

[15] Schuchat A, Team CC-R. Public Health Response to the Initiation and Spread of Pandemic COVID-19 in the United States, February 24-April 21, 2020. Morb. Mortal. Wkly. Rep. 69(18), 551–556 (2020). 10.15585/mmwr.mm6918e2 PMC7737947 32379733

[16] Parashar UD, Anderson LJ. Severe acute respiratory syndrome: review and lessons of the 2003 outbreak. Int. J. Epidemiol. 33(4), 628–634 (2004). 15155694 10.1093/ije/dyh198 PMC7108628

[17] Seto WH, Tsang D, Yung RW Effectiveness of precautions against droplets and contact in prevention of nosocomial transmission of severe acute respiratory syndrome (SARS). Lancet 361(9368), 1519–1520 (2003). 12737864 10.1016/S0140-6736(03)13168-6 PMC7112437

[18] Gandhi M, Yokoe DS, Havlir DV. Asymptomatic transmission the achilles' heel of current strategies to control Covid-19. N. Engl. J. Med. 382(22), 2158–2160 (2020). 32329972 10.1056/NEJMe2009758 PMC7200054

[19] van Doremalen N, Bushmaker T, Morris DH Aerosol and surface stability of SARS-CoV-2 as compared with SARS-CoV-1. N. Engl. J. Med. 382(16), 1564–1567 (2020). 32182409 10.1056/NEJMc2004973 PMC7121658

[20] Li W, Shi Z, Yu M Bats are natural reservoirs of SARS-like coronaviruses. Science 310(5748), 676–679 (2005). 16195424 10.1126/science.1118391

[21] Huang C, Wang Y, Li X Clinical features of patients infected with 2019 novel coronavirus in Wuhan, China. Lancet 395(10223), 497–506 (2020). 31986264 10.1016/S0140-6736(20)30183-5 PMC7159299

[22] Kakodkar P, Kaka N, Baig MN. A comprehensive literature review on the clinical presentation, and management of the pandemic coronavirus disease 2019 (COVID-19). Cureus 12(4), e7560 (2020). 32269893 10.7759/cureus.7560 PMC7138423

[23] Wong HYF, Lam HYS, Fong AH Frequency and distribution of chest radiographic findings in COVID-19 positive patients. Radiology 296(2), E72–E78 (2019). 10.1148/radiol.2020201160 PMC7233401 32216717

[24] Higny J, Feye F, Foret F. COVID-19 pandemic: overview of protective-ventilation strategy in ARDS patients. Acta Clin. Belg. 2020, 1–3 (2020). 10.1080/17843286.2020.1761162 32340583

[25] Gu J, Han B, Wang J. COVID-19: gastrointestinal manifestations and potential fecal–oral transmission. Gastroenterology 158(6), 1518–1519 (2020). 32142785 10.1053/j.gastro.2020.02.054 PMC7130192

[26] Zhang C, Shi L, Wang FS. Liver injury in COVID-19: management and challenges. Lancet Gastroenterol. Hepatol. 5(5), 428–430 (2020). 32145190 10.1016/S2468-1253(20)30057-1 PMC7129165

[27] Adams DH, Hubscher SG. Systemic viral infections and collateral damage in the liver. Am. J. Pathol. 168(4), 1057–1059 (2006). 16565481 10.2353/ajpath.2006.051296 PMC1606546

[28] Parvez MK. COVID-19 and coronaviral hepatitis: evidence of collateral damage. Future Virol. 15(6), 325–329 (2020).

[29] Parvez MK. Gastrointestinal and hepatobiliary manifestations of COVID-19: potential implications for healthcare resource-deficient countries. Gastroenterol. Hepatol. Lett. 2, 7–11 (2020).

[30] Liu Y, Yang Y, Zhang C Clinical and biochemical indexes from 2019-nCoV infected patients linked to viral loads and lung injury. Sci. China Life Sci. 63(3), 364–374 (2020). 32048163 10.1007/s11427-020-1643-8 PMC7088566

[31] Fang L, Karakiulakis G, Roth M. Are patients with hypertension and diabetes mellitus at increased risk for COVID-19 infection? Lancet Respir. Med. 8(4), e21 (2020). 32171062 10.1016/S2213-2600(20)30116-8 PMC7118626

[32] Higny J, Feye F, Foret F. COVID-19 pandemic: overview of protective-ventilation strategy in ARDS patients. Acta Clin. Belg. 2020, 1–3 (2020). 10.1080/17843286.2020.1761162 32340583

[33] Ceraolo C, Giorgi FM. Genomic variance of the 2019-nCoV coronavirus. J. Med. Virol. 92(5), 522–528 (2020). 32027036 10.1002/jmv.25700 PMC7166773

[34] Laha S, Chakraborty J, Das S, Manna SK, Biswas S, Chatterjee R. Characterizations of SARS-CoV-2 mutational profile, spike protein stability and viral transmission. Infect. Genet. Evol. 85, 104445 (2020). 32615316 10.1016/j.meegid.2020.104445 PMC7324922

[35] Benvenuto D, Demir AB, Giovanetti M, Ciccozzi M, Cassone A. Evidence for mutations in SARS-CoV-2 Italian isolates potentially affecting virus transmission. J. Med. Virol. 92(10), 2232–2237 (2020). 32492183 10.1002/jmv.26104 PMC7300971

[36] Kozlovskaya L, Piniaeva A, Ignatyev G Isolation and phylogenetic analysis of SARS-CoV-2 variants collected in Russia during the COVID-19 outbreak. Int. J. Infect. Dis. 99, 40–46 (2020). 32721529 10.1016/j.ijid.2020.07.024 PMC7834223

[37] Sheikh JA, Singh J, Singh H Emerging genetic diversity among clinical isolates of SARS-CoV-2: lessons for today. Infect. Genet. Evol. 84, 104330 (2020). 32335334 10.1016/j.meegid.2020.104330 PMC7180377

[38] Licastro D, Rajasekharan S, Dal Monego S, Curcio F, de Rosa R. Isolation and full-length genome characterization of Sarscov-2 from covid-19 cases in northern Italy. J. Virol. 94, e00543–20 (2020). 32238585 10.1128/JVI.00543-20 PMC7269454

[39] Cleaveland S, Laurenson MK, Taylor LH. Diseases of humans and their domestic mammals: pathogen characteristics, host range and the risk of emergence. Phil. Trans. R. Soc. Lond. B Biol. Sci. 356, 991–999 (2011). 10.1098/rstb.2001.0889 PMC1088494 11516377

[40] Li Y, Yang X, Wang N SNPs or RNA modifications? Concerns on mutation-based evolutionary studies of SARS-CoV-2. PLoS ONE 15, e0238490 (2020). 32857808 10.1371/journal.pone.0238490 PMC7454988

[41] Gorbalenya AE, Baker SC, Baric RS The species Severe acute respiratory syndrome-related coronavirus: classifying 2019-nCoV and naming it SARS-CoV-2. Nat. Microbiol. 5, 536–544 (2020). 32123347 10.1038/s41564-020-0695-z PMC7095448

[42] Kumar S, Maurya VK, Prasad AK, Bhatt MLB, Saxena SK. Structural, glycosylation and antigenic variation between 2019 novel coronavirus (2019-nCoV) and SARS coronavirus (SARS-CoV). VirusDis. 31, 13–21 (2020). 10.1007/s13337-020-00571-5 PMC7085496 32206694

[43] Chan JF, Kok KH, Zhu Z Genomic characterization of the 2019 novel human-pathogenic coronavirus isolated from a patient with atypical pneumonia after visiting Wuhan. Emerg. Microbes Infect. 9, 221–236 (2020). 31987001 10.1080/22221751.2020.1719902 PMC7067204

[44] Walls AC, Park YJ, Tortorici MA, Wall A, McGuire AT, Veesler D. Structure, function, and antigenicity of the SARS-CoV-2 spike glycoprotein. Cell 181, 281–292 (2020). 32155444 10.1016/j.cell.2020.02.058 PMC7102599

[45] Coutard B, Valle C, de Lamballerie X, Canard B, Seidah NG, Decroly E. The spike glycoprotein of the new coronavirus 2019-nCoV contains a furin-like cleavage site absent in CoV of the same clade. Antiviral Res. 176, 104742 (2020). 32057769 10.1016/j.antiviral.2020.104742 PMC7114094

[46] Wan Y, Shang J, Graham R, Baric RS, Li F. Receptor recognition by the novel coronavirus from Wuhan: an analysis based on decade-long structural studies of SARS coronavirus. J. Virol. 94, e00127–20 (2020). 31996437 10.1128/JVI.00127-20 PMC7081895

[47] Wrapp D, Wang N, Corbett KS Cryo-EM structure of the 2019-nCoV spike in the prefusion conformation. Science 367, 1260–1263 (2020). 32075877 10.1126/science.abb2507 PMC7164637

[48] Guan Y, Zheng BJ, He YQ Isolation and characterization of viruses related to the SARS coronavirus from animals in southern China. Science 302, 276–278 (2003). 12958366 10.1126/science.1087139

[49] Li Q, Guan X, Wu P Early transmission dynamics in Wuhan, China, of novel coronavirus-infected pneumonia. N. Engl. J. Med. 382, 1199–1207 (2020). 31995857 10.1056/NEJMoa2001316 PMC7121484

[50] Tai W, He L, Zhang X Characterization of the receptor-binding domain (RBD) of 2019 novel coronavirus: implication for development of RBD protein as a viral attachment inhibitor and vaccine. Cell Mol. Immunol. 17, 613–620 (2020). 32203189 10.1038/s41423-020-0400-4 PMC7091888

[51] Walls AC, Park YJ, Tortorici MA, Wall A, McGuire AT, Veesler D. Structure, function, and antigenicity of the SARS-CoV-2 spike glycoprotein. Cell 181, 281–292 (2020). 32155444 10.1016/j.cell.2020.02.058 PMC7102599

[52] Cantuti-Castelvetri L, Ojha R, Pedro LD Neuropilin-1 facilitates SARS-CoV-2 cell entry and infectivity. Science 370, 856–860 (2020). 33082293 10.1126/science.abd2985 PMC7857391

[53] Klenk HD, Garten W. Host cell proteases controlling virus pathogenicity. Trends Microbiol. 2, 39–4 (1994). 8162439 10.1016/0966-842x(94)90123-6

[54] Shah A, Rashid F, Aziz A, Jan AU, Suleman M. Genetic characterization of structural and open reading Fram-8 proteins of SARS-CoV-2 isolates from different countries. Gene Rep. 21, 100886 (2020). 32954047 10.1016/j.genrep.2020.100886 PMC7487737

[55] Shen L, Dien Bard J, Biegel JA, Judkins AR, Gai X. Comprehensive genome analysis of 6,000 USA SARS-CoV-2 isolates reveals haplotype signatures and localized transmission patterns by state and by country. Front. Microbiol. 11, 573430 (2020). 33013809 10.3389/fmicb.2020.573430 PMC7509426

[56] Tan YJ. The Severe Acute Respiratory Syndrome (SARS)-coronavirus 3a protein may function as a modulator of the trafficking properties of the spike protein. Virol. J. 2, 5 (2020). 10.1186/1743-422X-2-5 PMC549520 15703085

[57] Varshney B, Agnihothram S, Tan YJ, Baric R, Lal SK. SARS coronavirus 3b accessory protein modulates transcriptional activity of RUNX1b. PLoS ONE 7, e29542 (2012). 22253733 10.1371/journal.pone.0029542 PMC3257236

[58] Minakshi R, Padhan K, Rani M, Khan N, Ahmad F, Jameel S. The SARS Coronavirus 3a protein causes endoplasmic reticulum stress and induces ligand-independent downregulation of the type 1 interferon receptor. PLoS ONE 4, e8342 (2009). 20020050 10.1371/journal.pone.0008342 PMC2791231

[59] Konno Y, Kimura I, Uriu K SARS-CoV-2 ORF3b is a potent interferon antagonist whose activity is increased by a naturally occurring elongation variant. Cell Rep. 32, 108185 (2020). 32941788 10.1016/j.celrep.2020.108185 PMC7473339

[60] Hachim A, Kavian N, Cohen CA ORF8 and ORF3b antibodies are accurate serological markers of early and late SARS-CoV-2 infection. Nat. Immunol. 21, 1293–1301 (2020). 32807944 10.1038/s41590-020-0773-7

[61] Su YCF, Anderson DE, Young BE. Discovery and genomic characterization of a 382-nucleotide deletion in ORF7b and ORF8 during the early evolution of SARS-CoV-2. mBio 11, e01610–01620 (2020). 32694143 10.1128/mBio.01610-20 PMC7374062

[62] Parvez MK, Jagirdar RM, Purty RS COVID-19 pandemic: understanding the emergence, pathogenesis and containment. World Acad. Sci. J. 2, 18 (2020).

[63] Au CH, Chan WS, Lam HY Genome sequences of SARS-CoV-2 strains detected in Hong Kong. Microbiol. Resour. Announc. 9, e00697–20 (2020). 32732237 10.1128/MRA.00697-20 PMC7393966

[64] Coccia M. Factors determining the diffusion of COVID-19 and suggested strategy to prevent future accelerated viral infectivity similar to COVID. Sci. Total Environ. 729, 138474 (2020). 32498152 10.1016/j.scitotenv.2020.138474 PMC7169901

[65] Yu IT, Li Y, Wong TW Evidence of airborne transmission of the severe acute respiratory syndrome virus. N. Engl. J. Med. 350, 1731–1739 (2004). 15102999 10.1056/NEJMoa032867

[66] Parvez MK. Gut feeling: the plausible fecal-oral transmission route of Covid19. J. Infect. Dis. Epidemiol. 6, 141–143 (2020).

[67] Anderson RM, Fraser C, Ghani AC Epidemiology, transmission dynamics and control of SARS: the 2002–2003 epidemic. Philos. Trans. R. Soc. Lond. B Biol. Sci. 359, 1091–1105 (2004). 15306395 10.1098/rstb.2004.1490 PMC1693389

[68] Hufnagel L, Brockmann D, Geisel T. Forecast and control of epidemics in a globalized world. Proc. Natl Acad. Sci. USA 101, 15124–15129 (2004). 15477600 10.1073/pnas.0308344101 PMC524041

[69] Eletreby R, Zhuang Y, Carley KM, Yagan O, Poor HV. The effects of evolutionary adaptations on spreading processes in complex networks. Proc. Natl Acad. Sci. USA 117, 5664–5670 (2020). 32123091 10.1073/pnas.1918529117 PMC7084153

[70] Coccia M. An index to quantify environmental risk of exposure to future epidemics of the COVID-19 and similar viral agents: theory and practice. Environ. Res. 191, 110155 (2020). 32871151 10.1016/j.envres.2020.110155 PMC7834384

